# 506 Return to Work After Burn: PROMs Identify Impact Factors Other Than ‘usual Suspects’

**DOI:** 10.1093/jbcr/iraf019.135

**Published:** 2025-04-01

**Authors:** Dale Edgar, Inge Spronk, Mark Fear, Fiona Wood

**Affiliations:** The University of Notre Dame Australia; Dutch Burns Foundation; University of Western Australia; University of Western Australia

## Abstract

**Introduction:**

After a burn, returning to work (RTW) is a crucial goal in rehab to restore financial stability and a sense of normality, purpose, social integration and quality of life. However, burn survivors face numerous obstacles getting back to work, including physical limitations, psychological distress, and social stigma. There also remains a subgroup who do not RTW. This study aimed to investigate RTW rate and identify patient-reported factors associated with no return to work within 12 months of burn.

**Methods:**

This retrospective cohort study included adult burn patients injured 2012 - 2022. Patient-reported outcomes (PROMs) were assessed at 4-6 weeks and 3, 6, and 12 months post-burn. Patients were neither students, nor retired and had completed at least one survey about RTW. Outcomes: PROMs focussing on RTW and work impairment were used: Sickness Impact Profile (SIP, work scale) and Burn-Specific Health Scale-Brief (BSHS-B, work domain). Data analyses: In addition to descriptive analyses, mixed-effects adjusted regression models were created to analyze the RTW rate, work impairment, and BSHS-B work domain over time.

**Results:**

A sample of 866 patients (21.6% state burn cohort) were included, with 84.6% engaged prior in work outside managing their home. Most were male (70.3%), with median age 36.5 years (IQR: 27.0-50.0) and 83.6% had surgery. The median %TBSA was 2.5% (IQR: 0.8-7.0%). Workplace injury involved 22.5% (n=195) of the worker cohort. Responders’ age and gender did not differ from the WA burn population. However, responders had: less comorbidities (p=0.017); higher %TBSA (p< 0.001); surgery more frequently (p< 0.0001); more multiple-location burns (p=0.023); and, increased workplace injuries (p< 0.001).

RTW rate and impairment: By 6-weeks post-burn, 38% had RTW, increasing to 90% by 12 months. The mean time to return was 40 days (SD 50 days). At one month, SIP showed 79.2% reported work impairment that declined: 61.3% at 6 weeks, 55.8% at 3 months, 48.4% at 6 months, and 50% at 12 months. Receiving more occupational therapy (OT) 1-3 months post burn was associated with reduced reported work impairment.

No RTW within 12m: Those unable to RTW had: longer hospital stays (12 vs 7 days, p=0.026) and more surgeries (2.5 vs 1 surgery; p=0.015) but no evidence of difference in %TBSA, age, or gender characteristics. Significant predictors of no RTW included: contact and electrical burns; non-metro residence; workplace injury; readmissions; prolonged wound healing; as well as psychology sessions in the first 6 weeks; and, OT ongoing 6-12 months post burn.

**Conclusions:**

Common, non-modifiable factors were confirmed as barriers to RTW as expected.

**Applicability of Research to Practice:**

Deploying PROMs, confirmed that vocational and physical therapy and early psychological support can reduce impairment and enhance the likelihood of return to work within the first year after a burn injury.

**Funding for the Study:**

Foundation funding

